# 2-(6-Benzoyl-2-oxo-1,3-benzothia­zol-3-yl)acetic acid

**DOI:** 10.1107/S160053680905329X

**Published:** 2009-12-16

**Authors:** Abdullah Aydın, Mehmet Akkurt, Tijen Önkol, Orhan Büyükgüngör, M. Fethi Şahin

**Affiliations:** a Department of Science Education, Faculty of Education, Kastamonu University, 37200 Kastamonu, Turkey; b Department of Physics, Faculty of Arts and Sciences, Erciyes University, 38039 Kayseri, Turkey; c Department of Pharmaceutical Chemistry, Faculty of Pharmacy, Gazi University, 06330 Ankara, Turkey; d Department of Physics, Faculty of Arts and Sciences, Ondokuz Mayıs University, 55139 Samsun, Turkey

## Abstract

In the title compound, C_16_H_11_NO_4_S, the nine-membered fused ring is nearly planar, with maximum deviations from the mean plane of −0.022 (1) Å for the N atom and 0.011 (1) Å for the S atom, and makes a dihedral angle of 53.56 (7)° with the phenyl ring. The crystal structure is stabilized by O—H⋯O and C—H⋯O hydrogen-bonding inter­actions.

## Related literature

For the pharmacological effects of 2-benzoxazolinone/benzothia­zolinone derivatives, see: Ünlü *et al.* (2003 ▶); Petrov *et al.* (1994 ▶). For the quantum-chemical calculations, see: Pople & Beveridge (1970 ▶).
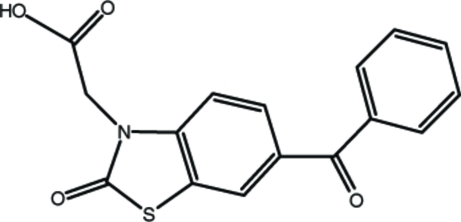


## Experimental

### 

#### Crystal data

C_16_H_11_NO_4_S
*M* *_r_* = 313.33
Orthorhombic, 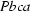

*a* = 11.4248 (3) Å
*b* = 8.9155 (2) Å
*c* = 27.6280 (8) Å
*V* = 2814.13 (13) Å^3^
*Z* = 8
Mo *K*α radiation
μ = 0.25 mm^−1^
*T* = 296 K
0.59 × 0.38 × 0.17 mm

#### Data collection

Stoe IPDS 2 diffractometer
Absorption correction: integration (*X-RED32*; Stoe & Cie, 2002 ▶) *T*_min_ = 0.868, *T*_max_ = 0.959
31503 measured reflections
3006 independent reflections
2363 reflections with *I* > 2σ(*I*)
*R*_int_ = 0.034

#### Refinement

*R*[*F* ^2^ > 2σ(*F* ^2^)] = 0.033
*wR*(*F* ^2^) = 0.084
*S* = 1.04
3006 reflections
199 parameters
H-atom parameters constrained
Δρ_max_ = 0.23 e Å^−3^
Δρ_min_ = −0.22 e Å^−3^

### 

Data collection: *X-AREA* (Stoe & Cie, 2002 ▶); cell refinement: *X-AREA*; data reduction: *X-RED32* (Stoe & Cie, 2002 ▶); program(s) used to solve structure: *SIR97* (Altomare *et al.*, 1999 ▶); program(s) used to refine structure: *SHELXL97* (Sheldrick, 2008 ▶); molecular graphics: *ORTEP-3 for Windows* (Farrugia, 1997 ▶); software used to prepare material for publication: *WinGX* (Farrugia, 1999 ▶).

## Supplementary Material

Crystal structure: contains datablocks global, I. DOI: 10.1107/S160053680905329X/hg2616sup1.cif

Structure factors: contains datablocks I. DOI: 10.1107/S160053680905329X/hg2616Isup2.hkl

Additional supplementary materials:  crystallographic information; 3D view; checkCIF report

## Figures and Tables

**Table 1:** Hydrogen-bond geometry (Å, °)

*D*—H⋯*A*	*D*—H	H⋯*A*	*D*⋯*A*	*D*—H⋯*A*
O4—H4*A*⋯O1^i^	0.82	1.86	2.6315 (18)	157
C9—H9⋯O2^ii^	0.93	2.43	3.1233 (19)	131
C15—H15*B*⋯O3^iii^	0.97	2.54	3.416 (2)	150

Symmetry codes: (i) 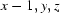; (ii) 
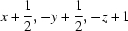
; (iii) 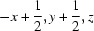.

**Fig. 1.**
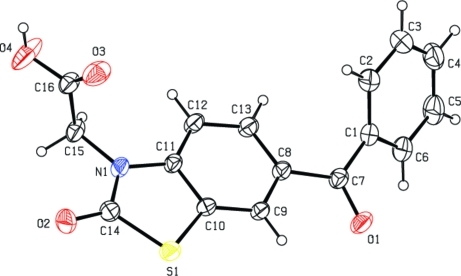
An ORTEP view of the title molecule with the atom numbering scheme. Displacement ellipsoids for non-H atoms are drawn at the 30% probability level.

**Fig. 2.**
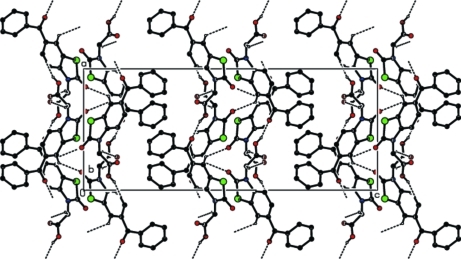
The packing and hydrogen bonding of the title compound viewed down b axis.Hydrogen atoms not involved in hydrogen bonding have been omitted for clarity.

**Fig. 3.**
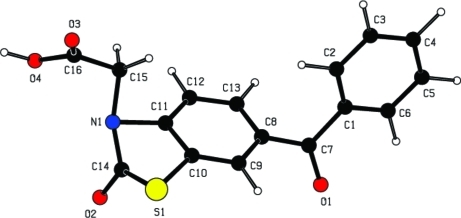
The spatial view of the title molecule (I), calculated by the CNDO aproximation.

## supplementary crystallographic information

### Comment

2-Benzoxazolinone / benzothiazolinone derivatives exhibit a variety of pharmacological effects, including anlgesic and anti-inflammatory activity (Ünlü *et al.*, 2003). It was studied analgesic and anti-inflammatory activity of the title compound which was synthesized by (Petrov *et al.*, 1994) previously. Based on this study, the title compound, (I), showed close anlgesic activity that of aspirin at 100 mg/kg dose, but in terms of anti-inflammatory activity, inactive (Ünlü *et al.*, 2003). In this study, the structure of the title compound (I) has been determined by single-crystal X-ray diffraction.

In (I) (Fig. 1), the nine-membered ring S1/N1/C8–C14 is nearly planar with maximum deviations of -0.022 (1) Å for N1 and 0.011 (1) Å for S1 from the mean plane. The dihedral angle between the nine-membered ring and the phenyl ring C1–C6 is 53.56 (7)° [the calculated value is 74.47°, using the *CNDO* (Pople *et al.*, 1970) aproximation]. The N1—C15—C16—O3 and N1—C15—C16—O4 torsion angles related with the carboxyl group are 0.7 (3) and -177.21 (15)° [the calculated values are 114.5° and -65.95°].

In the crystal structure of (I), there exist intermolecular O—H···O and C—H···O hydrogen bonding interactions (Table 1, Fig. 2).

The quantum-chemical calculation of (I) was carried out by using the *CNDO* aproximation. The HOMO and LUMO energy levels of (I) are -10.4385 and 2.0553 eV, respectively. Its calculated molecule dipole moment is 8.206 Debye (1 D = 3.33564 × 10 ^-30^ C.m.). Due to the intermolecular interactions in the crystal structure of (I), the spatial configurations obtained by the theoretical *CNDO* and experimental X-rays for (I) are almost different (see Figs. 1 & 3). But the geometric parameters in (I) are almost comparable within the experimental error interval in the results of both methods.

### Experimental

Methyl (6-benzoyl-2-oxo-1,3-benzothiazol-3-yl)acetate (2 mmol) and sodium hydroxide (2 mmol) in 30 ml e thanol/water (25:5) was refluxed for 4 h. After cooling to room temperature, the mixture was acidified with 1 N HCl (30 ml) to give a solid precipitate.The product was collected by suction filtration, washed with water, dried, and crystallized from ethanol/water to yield % 73 [m.p.: 524 K].

### Refinement

H atoms were placed geometrically, with O—H = 0.82 Å, C—H = 0.93–0.97 Å, and treated using a riding model, with *U*_iso_(H) = 1.2*U*_eq_(C) or 1.5*U*_eq_(O).

### Crystal data

**Table:**

C_16_H_11_NO_4_S	*F*(000) = 1296
*M**_r_* = 313.33	*D*_x_ = 1.479 Mg m^−^^3^
Orthorhombic, *P**b**c**a*	Mo *K*α radiation, λ = 0.71073 Å
Hall symbol: -P 2ac 2ab	Cell parameters from 31743 reflections
*a* = 11.4248 (3) Å	θ = 1.5–27.3°
*b* = 8.9155 (2) Å	µ = 0.25 mm^−^^1^
*c* = 27.6280 (8) Å	*T* = 296 K
*V* = 2814.13 (13) Å^3^	Prismatic stick, colourless
*Z* = 8	0.59 × 0.38 × 0.17 mm

### Data collection

**Table:**

Stoe IPDS 2 diffractometer	3006 independent reflections
Radiation source: sealed X-ray tube, 12 x 0.4 mm long-fine focus	2363 reflections with *I* > 2σ(*I*)
plane graphite	*R*_int_ = 0.034
Detector resolution: 6.67 pixels mm^-1^	θ_max_ = 26.8°, θ_min_ = 1.5°
ω scans	*h* = −14→14
Absorption correction: integration (*X-RED32*; Stoe & Cie, 2002)	*k* = −11→11
*T*_min_ = 0.868, *T*_max_ = 0.959	*l* = −34→34
31503 measured reflections	

### Refinement

**Table:**

Refinement on *F*^2^	Primary atom site location: structure-invariant direct methods
Least-squares matrix: full	Secondary atom site location: difference Fourier map
*R*[*F*^2^ > 2σ(*F*^2^)] = 0.033	Hydrogen site location: inferred from neighbouring sites
*wR*(*F*^2^) = 0.084	H-atom parameters constrained
*S* = 1.04	*w* = 1/[σ^2^(*F*_o_^2^) + (0.0454*P*)^2^ + 0.2486*P*] where *P* = (*F*_o_^2^ + 2*F*_c_^2^)/3
3006 reflections	(Δ/σ)_max_ = 0.001
199 parameters	Δρ_max_ = 0.23 e Å^−^^3^
0 restraints	Δρ_min_ = −0.22 e Å^−^^3^

### Special details

**Table:**

Geometry. Bond distances, angles *etc*. have been calculated using the rounded fractional coordinates. All su's are estimated from the variances of the (full) variance-covariance matrix. The cell e.s.d.'s are taken into account in the estimation of distances, angles and torsion angles
Refinement. Refinement on *F*^2^ for ALL reflections except those flagged by the user for potential systematic errors. Weighted *R*-factors *wR* and all goodnesses of fit *S* are based on *F*^2^, conventional *R*-factors *R* are based on *F*, with *F* set to zero for negative *F*^2^. The observed criterion of *F*^2^ > σ(*F*^2^) is used only for calculating -*R*-factor-obs *etc*. and is not relevant to the choice of reflections for refinement. *R*-factors based on *F*^2^ are statistically about twice as large as those based on *F*, and *R*-factors based on ALL data will be even larger.

### Fractional atomic coordinates and isotropic or equivalent isotropic displacement parameters (Å^2^)

**Table:**

	*x*	*y*	*z*	*U*_iso_*/*U*_eq_	
S1	0.57030 (3)	0.23072 (5)	0.52487 (1)	0.0462 (1)	
O1	0.92276 (8)	0.45469 (15)	0.63203 (5)	0.0573 (4)	
O2	0.35095 (10)	0.25597 (15)	0.49337 (5)	0.0616 (4)	
O3	0.22769 (11)	0.33284 (18)	0.61382 (5)	0.0748 (5)	
O4	0.11738 (11)	0.5146 (2)	0.58490 (7)	0.1006 (7)	
N1	0.41141 (9)	0.41838 (15)	0.55272 (5)	0.0415 (4)	
C1	0.85487 (12)	0.61124 (17)	0.69378 (6)	0.0431 (5)	
C2	0.78137 (14)	0.6046 (2)	0.73373 (6)	0.0551 (6)	
C3	0.80669 (18)	0.6877 (3)	0.77475 (7)	0.0681 (7)	
C4	0.90272 (18)	0.7800 (3)	0.77570 (8)	0.0700 (8)	
C5	0.97516 (18)	0.7884 (2)	0.73637 (8)	0.0683 (7)	
C6	0.95280 (14)	0.7034 (2)	0.69545 (7)	0.0536 (6)	
C7	0.83707 (12)	0.51557 (17)	0.65028 (6)	0.0413 (5)	
C8	0.71999 (11)	0.49170 (17)	0.62847 (5)	0.0377 (4)	
C9	0.71055 (11)	0.38169 (17)	0.59286 (5)	0.0381 (4)	
C10	0.60499 (11)	0.36100 (17)	0.56983 (5)	0.0369 (4)	
C11	0.50840 (11)	0.45213 (17)	0.58098 (5)	0.0368 (4)	
C12	0.51704 (12)	0.56159 (18)	0.61601 (6)	0.0439 (5)	
C13	0.62268 (12)	0.58068 (18)	0.63966 (6)	0.0421 (5)	
C14	0.42573 (13)	0.30165 (19)	0.52075 (6)	0.0450 (5)	
C15	0.30209 (11)	0.5004 (2)	0.55357 (6)	0.0467 (5)	
C16	0.21427 (12)	0.4378 (2)	0.58824 (6)	0.0474 (5)	
H2	0.71500	0.54420	0.73290	0.0660*	
H3	0.75840	0.68090	0.80180	0.0820*	
H4	0.91860	0.83690	0.80310	0.0840*	
H4A	0.06890	0.48070	0.60390	0.1510*	
H5	1.03990	0.85160	0.73710	0.0820*	
H6	1.00330	0.70810	0.66910	0.0640*	
H9	0.77490	0.32290	0.58480	0.0460*	
H12	0.45300	0.62150	0.62360	0.0530*	
H13	0.62920	0.65400	0.66350	0.0500*	
H15A	0.26860	0.49990	0.52130	0.0560*	
H15B	0.31800	0.60390	0.56220	0.0560*	

### Atomic displacement parameters (Å^2^)

**Table:**

	*U*^11^	*U*^22^	*U*^33^	*U*^12^	*U*^13^	*U*^23^
S1	0.0364 (2)	0.0550 (2)	0.0471 (2)	−0.0031 (2)	0.0034 (2)	−0.0099 (2)
O1	0.0299 (5)	0.0725 (8)	0.0696 (8)	0.0023 (5)	0.0037 (5)	−0.0135 (7)
O2	0.0450 (6)	0.0859 (9)	0.0539 (7)	−0.0120 (6)	−0.0099 (5)	−0.0088 (7)
O3	0.0626 (8)	0.0835 (10)	0.0784 (9)	0.0153 (7)	0.0236 (7)	0.0317 (8)
O4	0.0414 (7)	0.1141 (13)	0.1464 (15)	0.0268 (8)	0.0339 (8)	0.0644 (12)
N1	0.0271 (5)	0.0520 (8)	0.0453 (7)	−0.0015 (5)	0.0000 (5)	0.0024 (6)
C1	0.0366 (7)	0.0452 (9)	0.0474 (8)	0.0017 (6)	−0.0073 (6)	0.0022 (7)
C2	0.0472 (9)	0.0682 (12)	0.0500 (10)	0.0000 (8)	−0.0019 (7)	0.0012 (9)
C3	0.0670 (11)	0.0905 (15)	0.0468 (10)	0.0117 (11)	−0.0050 (8)	−0.0069 (10)
C4	0.0698 (12)	0.0787 (15)	0.0614 (12)	0.0119 (10)	−0.0237 (10)	−0.0187 (11)
C5	0.0575 (10)	0.0647 (13)	0.0827 (15)	−0.0073 (9)	−0.0250 (10)	−0.0094 (11)
C6	0.0413 (8)	0.0592 (10)	0.0604 (11)	−0.0045 (7)	−0.0089 (7)	−0.0012 (9)
C7	0.0323 (7)	0.0457 (9)	0.0460 (8)	−0.0017 (6)	0.0014 (6)	0.0029 (7)
C8	0.0308 (6)	0.0420 (8)	0.0402 (8)	−0.0007 (6)	0.0016 (5)	0.0008 (7)
C9	0.0294 (6)	0.0442 (8)	0.0408 (8)	0.0031 (6)	0.0041 (5)	0.0007 (7)
C10	0.0307 (6)	0.0420 (8)	0.0379 (7)	−0.0018 (5)	0.0048 (5)	0.0011 (6)
C11	0.0269 (6)	0.0431 (8)	0.0405 (8)	−0.0010 (5)	0.0019 (5)	0.0046 (7)
C12	0.0313 (7)	0.0473 (9)	0.0532 (9)	0.0070 (6)	0.0031 (6)	−0.0044 (8)
C13	0.0365 (7)	0.0422 (8)	0.0476 (9)	0.0014 (6)	0.0014 (6)	−0.0064 (7)
C14	0.0359 (7)	0.0580 (10)	0.0410 (8)	−0.0095 (7)	0.0021 (6)	0.0037 (7)
C15	0.0290 (6)	0.0546 (9)	0.0564 (9)	0.0015 (6)	−0.0007 (6)	0.0095 (8)
C16	0.0340 (7)	0.0529 (10)	0.0552 (10)	0.0027 (6)	0.0037 (6)	0.0047 (8)

### Geometric parameters (Å, °)

**Table:**

S1—C10	1.7462 (15)	C8—C13	1.400 (2)
S1—C14	1.7723 (16)	C8—C9	1.393 (2)
O1—C7	1.2277 (18)	C9—C10	1.3760 (18)
O2—C14	1.212 (2)	C10—C11	1.4046 (19)
O3—C16	1.183 (2)	C11—C12	1.378 (2)
O4—C16	1.305 (2)	C12—C13	1.383 (2)
O4—H4A	0.8200	C15—C16	1.495 (2)
N1—C14	1.375 (2)	C2—H2	0.9300
N1—C15	1.4475 (18)	C3—H3	0.9300
N1—C11	1.3885 (18)	C4—H4	0.9300
C1—C6	1.389 (2)	C5—H5	0.9300
C1—C7	1.488 (2)	C6—H6	0.9300
C1—C2	1.388 (2)	C9—H9	0.9300
C2—C3	1.385 (3)	C12—H12	0.9300
C3—C4	1.372 (3)	C13—H13	0.9300
C4—C5	1.368 (3)	C15—H15A	0.9700
C5—C6	1.385 (3)	C15—H15B	0.9700
C7—C8	1.4824 (19)		
			
S1···N1	2.5858 (13)	C15···O3^viii^	3.416 (2)
S1···O2^i^	3.2479 (12)	C15···O2^viii^	3.319 (2)
S1···C15^ii^	3.5452 (17)	C1···H13	2.7400
S1···O4^iii^	3.3258 (17)	C2···H13	2.6400
S1···H15B^ii^	3.1000	C4···H2^xii^	2.9600
O1···O4^iv^	2.6315 (18)	C7···H4A^iv^	2.9600
O1···C13^v^	3.381 (2)	C8···H2	2.9200
O2···S1^vi^	3.2479 (12)	C12···H15B	2.7400
O2···C15^iii^	3.319 (2)	C13···H9^xii^	2.8900
O2···C9^vi^	3.1233 (19)	C13···H2	2.8000
O3···N1	2.7994 (18)	C15···H12	2.8100
O3···C15^iii^	3.416 (2)	C16···H15B^iii^	3.0800
O3···C3^vii^	3.363 (3)	H2···C8	2.9200
O4···C14^viii^	3.152 (2)	H2···C13	2.8000
O4···S1^viii^	3.3258 (17)	H2···H13	2.3700
O4···O1^ix^	2.6315 (18)	H2···C4^v^	2.9600
O1···H9	2.4400	H3···O3^xi^	2.7000
O1···H13^v^	2.8800	H4···O1^xiii^	2.7600
O1···H6	2.6500	H4···O3^xi^	2.8400
O1···H4A^iv^	1.8600	H4A···O1^ix^	1.8600
O1···H4^x^	2.7600	H4A···C7^ix^	2.9600
O2···H15A^iii^	2.7700	H6···O1	2.6500
O2···H15A	2.4900	H9···O1	2.4400
O2···H9^vi^	2.4300	H9···O2^i^	2.4300
O3···H4^vii^	2.8400	H9···C13^v^	2.8900
O3···H15B^iii^	2.5400	H12···C15	2.8100
O3···H12^iii^	2.8100	H12···H15B	2.3000
O3···H3^vii^	2.7000	H12···O3^viii^	2.8100
N1···S1	2.5858 (13)	H13···C1	2.7400
N1···O3	2.7994 (18)	H13···C2	2.6400
C2···C13	3.176 (2)	H13···H2	2.3700
C3···O3^xi^	3.363 (3)	H13···O1^xii^	2.8800
C9···C13^v^	3.536 (2)	H15A···O2	2.4900
C9···O2^i^	3.1233 (19)	H15A···O2^viii^	2.7700
C13···C2	3.176 (2)	H15B···C12	2.7400
C13···O1^xii^	3.381 (2)	H15B···H12	2.3000
C13···C9^xii^	3.536 (2)	H15B···S1^ii^	3.1000
C14···O4^iii^	3.152 (2)	H15B···O3^viii^	2.5400
C15···S1^ii^	3.5452 (17)	H15B···C16^viii^	3.0800
			
C10—S1—C14	91.14 (7)	S1—C14—N1	109.86 (11)
C16—O4—H4A	110.00	O2—C14—N1	124.87 (14)
C11—N1—C14	115.49 (12)	S1—C14—O2	125.26 (13)
C11—N1—C15	124.75 (13)	N1—C15—C16	113.63 (14)
C14—N1—C15	119.71 (12)	O3—C16—C15	126.24 (14)
C2—C1—C7	122.36 (13)	O4—C16—C15	109.15 (15)
C6—C1—C7	118.43 (14)	O3—C16—O4	124.57 (16)
C2—C1—C6	119.09 (16)	C1—C2—H2	120.00
C1—C2—C3	120.11 (16)	C3—C2—H2	120.00
C2—C3—C4	120.25 (18)	C2—C3—H3	120.00
C3—C4—C5	120.1 (2)	C4—C3—H3	120.00
C4—C5—C6	120.46 (19)	C3—C4—H4	120.00
C1—C6—C5	119.97 (17)	C5—C4—H4	120.00
O1—C7—C8	119.28 (14)	C4—C5—H5	120.00
C1—C7—C8	122.28 (12)	C6—C5—H5	120.00
O1—C7—C1	118.44 (13)	C1—C6—H6	120.00
C7—C8—C9	117.25 (12)	C5—C6—H6	120.00
C9—C8—C13	119.55 (12)	C8—C9—H9	120.00
C7—C8—C13	123.05 (13)	C10—C9—H9	120.00
C8—C9—C10	119.26 (13)	C11—C12—H12	121.00
S1—C10—C11	111.25 (10)	C13—C12—H12	121.00
C9—C10—C11	120.64 (13)	C8—C13—H13	119.00
S1—C10—C9	128.10 (11)	C12—C13—H13	119.00
N1—C11—C10	112.23 (13)	N1—C15—H15A	109.00
N1—C11—C12	127.27 (13)	N1—C15—H15B	109.00
C10—C11—C12	120.49 (12)	C16—C15—H15A	109.00
C11—C12—C13	118.78 (13)	C16—C15—H15B	109.00
C8—C13—C12	121.25 (15)	H15A—C15—H15B	108.00
			
C14—S1—C10—C9	−178.35 (15)	C2—C3—C4—C5	−1.1 (4)
C14—S1—C10—C11	0.72 (12)	C3—C4—C5—C6	−0.4 (3)
C10—S1—C14—N1	0.32 (12)	C4—C5—C6—C1	1.4 (3)
C10—S1—C14—O2	179.26 (16)	O1—C7—C8—C13	−165.40 (15)
C11—N1—C15—C16	−92.11 (18)	C1—C7—C8—C9	−169.92 (14)
C15—N1—C14—O2	−2.9 (2)	C1—C7—C8—C13	14.6 (2)
C11—N1—C14—S1	−1.33 (17)	O1—C7—C8—C9	10.1 (2)
C15—N1—C11—C12	3.7 (2)	C9—C8—C13—C12	0.0 (2)
C14—N1—C11—C12	−179.09 (15)	C7—C8—C13—C12	175.37 (15)
C11—N1—C14—O2	179.73 (16)	C7—C8—C9—C10	−176.64 (13)
C14—N1—C11—C10	1.91 (19)	C13—C8—C9—C10	−1.0 (2)
C14—N1—C15—C16	90.74 (18)	C8—C9—C10—C11	1.8 (2)
C15—N1—C14—S1	176.08 (11)	C8—C9—C10—S1	−179.27 (11)
C15—N1—C11—C10	−175.35 (14)	S1—C10—C11—C12	179.35 (12)
C7—C1—C6—C5	−176.84 (16)	C9—C10—C11—N1	177.56 (13)
C2—C1—C7—C8	43.9 (2)	S1—C10—C11—N1	−1.58 (16)
C2—C1—C6—C5	−0.8 (2)	C9—C10—C11—C12	−1.5 (2)
C6—C1—C7—C8	−140.18 (16)	C10—C11—C12—C13	0.5 (2)
C6—C1—C7—O1	39.8 (2)	N1—C11—C12—C13	−178.44 (15)
C7—C1—C2—C3	175.14 (17)	C11—C12—C13—C8	0.3 (2)
C2—C1—C7—O1	−136.10 (17)	N1—C15—C16—O3	0.7 (3)
C6—C1—C2—C3	−0.8 (3)	N1—C15—C16—O4	−177.21 (15)
C1—C2—C3—C4	1.7 (3)		

Symmetry codes: (i) *x*+1/2, −*y*+1/2, −*z*+1; (ii) −*x*+1, −*y*+1, −*z*+1; (iii) −*x*+1/2, *y*−1/2, *z*; (iv) *x*+1, *y*, *z*; (v) −*x*+3/2, *y*−1/2, *z*; (vi) *x*−1/2, −*y*+1/2, −*z*+1; (vii) −*x*+1, *y*−1/2, −*z*+3/2; (viii) −*x*+1/2, *y*+1/2, *z*; (ix) *x*−1, *y*, *z*; (x) −*x*+2, *y*−1/2, −*z*+3/2; (xi) −*x*+1, *y*+1/2, −*z*+3/2; (xii) −*x*+3/2, *y*+1/2, *z*; (xiii) −*x*+2, *y*+1/2, −*z*+3/2.

### Hydrogen-bond geometry (Å, °)

**Table:**

*D*—H···*A*	*D*—H	H···*A*	*D*···*A*	*D*—H···*A*
O4—H4A···O1^ix^	0.82	1.86	2.6315 (18)	157
C9—H9···O2^i^	0.93	2.43	3.1233 (19)	131
C15—H15B···O3^viii^	0.97	2.54	3.416 (2)	150

Symmetry codes: (ix) *x*−1, *y*, *z*; (i) *x*+1/2, −*y*+1/2, −*z*+1; (viii) −*x*+1/2, *y*+1/2, *z*.
